# Clinical Classifications of Children With Psychogenic Non-epileptic Seizure

**DOI:** 10.3389/fped.2020.596781

**Published:** 2021-01-25

**Authors:** Li-Ping Zhang, Yu Jia, Hao Huang, Da-Wei Li, Yu-Ping Wang

**Affiliations:** ^1^Department of Pediatric, Xuanwu Hospital, Capital Medical University, Beijing, China; ^2^Department of Neurology, Xuanwu Hospital, Capital Medical University, Beijing, China; ^3^Medical Records and Statistics Department, Xuanwu Hospital, Capital Medical University, Beijing, China

**Keywords:** psychogenic non-epileptic seizure, epilepsy, Chinese pediatric population, semiogical classification, diagnosis

## Abstract

**Objective:** To analyze the clinical features of children with psychogenic non-epileptic seizures in one tertiary center in China.

**Methods:** Clinical data including medical records and video- electroencephalograph (video-EEG) monitoring records of 88 pediatric PNES patients hospitalized in the pediatric department of Xuanwu Hospital, Beijing, China from April, 2012 to April, 2018 were collected in this study. Demographic information of patients, semiological classification, duration, and frequency of symptoms, risk factors as well as comorbidity were summarized and analyzed.

**Results:** For semiological classification, all PNES related symptoms were divided into different categories: motor symptoms, unresponsiveness, sensory symptoms, visceral symptoms, and abnormal behaviors, among which motor symptoms were the most prevalent form. Risk factors were reviewed and categorized into two groups: persistent factors and predisposing factors, and patients were most frequently affected by the influences of families. The duration and frequency of symptoms varied substantially within PNES patients while the average time of duration was relatively longer than epilepsy as reported previously. Epilepsy was considered as the most frequent comorbidity of PNES and PNES patients misdiagnosed as epilepsy often mistreated with antiseizure medication.

**Significance:** Our study showed that motor PNES are the most frequent seizure type. Family issues were a risk factor for PNES. Epilepsy was the most frequent co-existing neurological comorbidity.

## Introduction

Psychogenic non-epileptic seizure (PNES) is a common disorder characterized by seizure-like symptoms and other neurobehavioral dysfunctions, which are also identified within epilepsy patients (1). Unlike epilepsy, however, PNES is originated psychologically and does not associate with abnormal cortical electrical discharges (2). In the past, PNES patients were often misdiagnosed as epilepsy and treated with antiseizure medication (ASM), which impact patients' health due to unnecessary drug intake and delayed diagnosis (3). Therefore, it is important to develop accurate diagnosing strategies at the initial stage of the disease.

Despite the fact that children are also severely affected by PNES, as compared with adults, fewer studies focusing on PNES diagnosis in the pediatric population have been carried out (4, 5). It was estimated that the prevalence of PNES in the adult is 2–33/100,000 and would be lower in children (6, 7). In other studies, 3.5–20% of children and adolescents undergoing Video EEG monitoring (VEEG) were diagnosed as PNES (8–10). With early therapeutic interventions, most children/adolescents regained normal functions eventually (11, 12). These results suggest the importance and significance of diagnosing and treating PNES in children.

VEEG has been the most commonly used technique in PNES diagnosis as it is straightforward to distinguish epilepsy from non-epilepsy seizures by detecting abnormal electrical activity in the brain (1, 2). In clinical settings, VEEG is usually utilized in combination with semiological classifications for PNES diagnosis (8, 10). In recent years, a growing number of studies have established/modified the framework for PNES semiology, while a consistent conclusion has not been formed yet (8, 10, 13).

Previous studies have categorized the symptoms of childhood PNES with a series of clinical characteristics ranging from movement to emotional disorders, which highlighted the importance of accurate semiological classification in the early diagnosis of PNES in children (4, 8, 11). In our clinical practice, pediatric patients diagnosed with PNES experienced similar, while not exactly the same symptoms compared with what described previously (8, 10, 13), particularly, movement disorders (e.g., limb, trunk, eyes), unresponsive (e.g., pseudosyncope), sensory symptoms (e.g., headache, numbness), visceral symptoms (e.g., nausea, abdominal pain), and abnormal behaviors were five of the most commonly seen types of symptoms, unfortunately, some of them have not been fully characterized by far.

In the present study, we retrospectively analyzed the medical records and VEEG records of Chinese pediatric patients diagnosed with PNES in our hospital in the past 6 years. Clinical symptoms were classified into five major groups as above and the prevalence of each group was calculated, the duration and frequency of symptom onsets, risk factors as well as comorbidity were analyzed. Our study aims to provide new insights regarding the major clinical features of children with psychogenic non-epileptic seizures in one tertiary center in China.

## Methods

### Patients

From April 2012 to April 2018, pediatric patients (younger than 18 years old) who experienced seizure-like symptoms were admitted to the pediatric neurological ward of Xuanwu Hospital, Beijing, China. Demographical information including gender, age, place of residence, family status, medication history was obtained before VEEG. Patients were diagnosed as PNES based on VEEG and clinical data meet all the following criteria (14): (1) Sudden onset of symptoms with no abnormal electrical signal detected by VEEG within the same period; (2) No clinical evidence for an alternative diagnosis (epilepsy or other neurological disorders than PNES); (3) At least one typical event was captured by VEEG (for symptoms with low frequency, video records provided by patients' parents were used in combination with VEEG for diagnosis). Besides, patients were considered as non-PNES according to each of the following criteria: (1) Remain inconclusive according to VEEG records and clinical symptoms; (2) The mismatch between VEEG recorded symptoms and patient-reported symptoms. (3) Only subjective symptoms were reported. PNES patients who also have other recognized paroxysmal behavioral episodes, with clear clinical, electroencephalographic, and imaging evidence suggestive of coexisting real epileptic seizures, supported by VEEG results, were also included for analysis. Written informed consent was obtained from each patient or the patient's parent(s) before VEEG. Our study was authorized by the ethical committee of Xuanwu Hospital.

### Video EEG Monitoring

PN-NET synchronous video-electroencephalogram-topography instrument was from Beijing Yun-Shen Technology Co. LTD (Beijing). All patients had continuous inpatient VEEG for an average period of 16 h. Scalp electrodes were placed in accordance with the 10–20 international electrode system. Patients' responsiveness and reactivity were tested during and after the attacks. EEG and audio-visual signals were acquired and analyzed by at least two specialists who were unaware of the study.

### Semiology

The semiology of clinical events was visually analyzed based on previous classifications and our clinical experience (8, 10, 13). Specifically, 5 categories were established including (1) Motor symptoms. Dysfunctional or abnormal movements of the face, limbs, and trunk.; (2) Unresponsiveness. No response to external stimuli, little facial expressions or body movements, or even pseudo syncope; (3) Sensory symptoms. Neural perception dysfunction with somatic symptoms, represented by headache, dizziness, limb numbness, and paroxysmal unclear vision; (4) Visceral symptoms. Abdominal and chest pain/uncomfortable. (5) Abnormal behaviors. Other unusual behaviors were similar to psychological symptoms. Each symptom captured by VEEG was classified into each of the above categories. For patients with more than one captured category/event, every single category in each event was analyzed independently.

### Symptom Duration and Frequency Analysis

Symptom duration as well as the frequency of symptoms were recorded and analyzed during and/or after VEEG. To analyze the time of symptoms durations, both the longest and shortest symptoms durations for each semiology category of each patient were recorded and compared with other patients' data, respectively. For frequency analysis, the time of interval between two adjacent symptom onsets (of each semiology category) was analyzed and was divided into 3 groups: mild (several times per year), moderate (several times per month), and severe (daily or several times per day).

### Risk Factor and Comorbidity Analysis

Risk factors and comorbidity of PNES were analyzed according to medical records and self-reports provided by patients and/or their parents. Specifically, trauma, physical and/or mental abuse, and previous history of epilepsy or other diseases were investigated in follow-up interviews and patient file reviews.

### Statistics

Statistical analysis was done by GraphPad Prism (version 8). For analyzing the duration of symptoms, median, minimum, maximum, 25% percentile, and 75% percentile were calculated.

## Results

### Demographics

Among 4,268 children patients with seizure-like symptoms, 88 were diagnosed as PNES and included in our study. Fifty-four cases were boys (61%), and 34 cases were girls (39%). The age of children at VEEG was five-fourteen years (mean 10.41 ± 2.04). Twenty-three cases (26%) were from the urban area and sixty-five cases (74%) from the rural area. Thirty-two patients (36%) were from one-child families and fifty-six patients (64%) were from multiple-children families (Table 1).

**Table 1:** Demographic information of patients.

Gender	Boy (cases)	Boy (%)	Girl (cases)	Girl (%)
	54	61	34	39
Living area	Rural (cases)	Rural (%)	Urban (cases)	Urban (%)
	23	26	65	74
Siblings	With (cases)	With (%)	Without (%)	Without (%)
	32	36	56	64
Age	Minimum (year)	Maximum (year)	Median ± SD (year)	
	5	14	10.41 ± 2.04	

### Semiology

As described above, VEEG records of each patient were analyzed in combination with medical records. The majority (eighty-two cases, 93%) of patients were awake during the onset of symptoms, and the rest (six cases, 7%) were asleep. A total of 143 typical PNES events were observed and were classified into following categories (Table 2): fifty-five motor symptoms (38%), twenty-five unresponsiveness (18%), thirty-nine sensory symptoms (27%), nine visceral symptoms (6%), and fifteen abnormal behaviors (10%).

**Table 2:** Semiological classification.

	**Events observed**	**Percentage (%)**
**Motor symptoms**	55	38
Head shaking	2	1
Limb shaking	17	12
Body shaking	2	1
Mouth spasms	2	1
Limb spasms	8	6
Abnormal eye movements	14	10
Limb stiffness	6	4
Clenched fists	4	3
**Unresponsiveness**	25	18
**Sensory symptoms**	39	27
Headache	19	13
Dizziness	10	7
Limb numbness	6	4
Paroxysmal blurred vision	4	3
**Visceral symptoms**	9	6
Abdominal pain	3	2
Abdominal discomfort	3	2
Nausea/vomiting	1	1
Chest pain	2	1
**Abnormal behaviors**	15	10
Crying	3	2
Shouting	2	1
Disorganized speech/aphasia	6	4
Daze	2	1
Laughing	2	1

*PNES related symptoms were classified into 5 categories as indicated, number of events as well as percentage of each category was calculated (percentage values rounded to nearest whole number)*.

**Figure 1:**
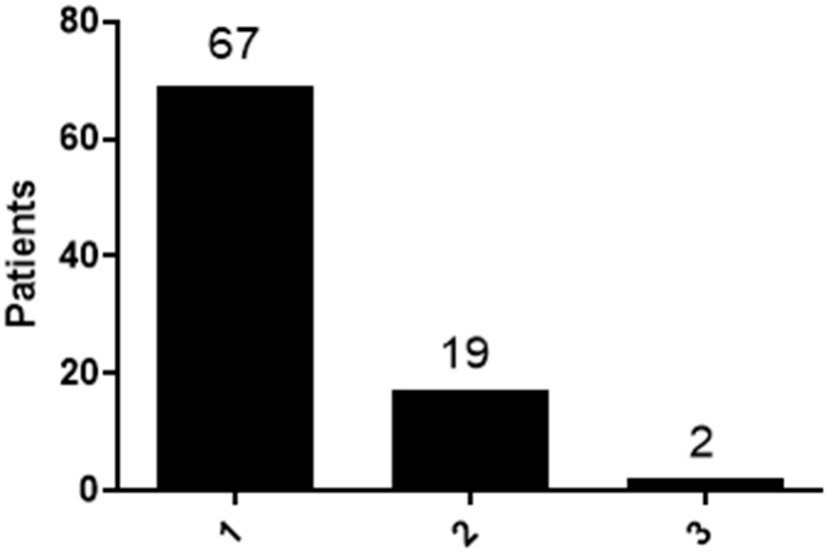
Distribution of patients with different type of semiology categories. Patients with 1 category, 2 categories and 3 categories were calculated and the distribution was shown.

In total of eighty-eight patients, sixty-nine had symptoms within one semiology category (78%), while seventeen patients (19%) had two categories of PNES events including: four patients had both motor symptoms and unresponsiveness; two patients had both sensory symptoms and unresponsiveness; three patients had both motor symptoms and sensory symptoms; three patients had both unresponsiveness and visceral symptoms; one had both motor symptoms and visceral symptoms; two patients had both motor symptoms and abnormal behaviors; one patient had both sensory symptoms and abnormal behaviors; one patient had both visceral symptoms and abnormal behaviors. Furthermore, two patients (2%) had three different semiology categories including one patient had motor symptoms, unresponsiveness and sensory symptoms, one patient had motor symptoms, unresponsiveness and abnormal behaviors (Figure 1).

Motor symptoms include dysfunctional or abnormal movements of the face, limbs, and trunk. Tremor is the most frequently identified sign in patients with motor symptoms, which include two head shaking, seventeen limbs shaking and two body shaking. Spasms are also frequently observed, including two mouth spasms and eight limb spasms. Besides, fourteen abnormal eye movements, six limb stiffness and four clenched fists cases were observed (Table 2).

Patients with unresponsiveness showed no response to external stimuli, little facial expressions or body movements, or even pseudo syncope (Table 2).

Sensory symptoms were caused by abnormalities in neural perception and were represented by headache, dizziness, limb numbness, and paroxysmal unclear vision. In patients with sensory symptoms, nineteen headaches, ten dizziness, six limb numbness and four paroxysmal blurred vision cases were observed (Table 2).

Visceral symptom was manifested as abdominal pain, abdominal discomfort, nausea/vomiting, and chest pain. In patients with visceral symptoms, three abdominal pain, three abdominal discomfort, one nausea/vomiting, and two chest pain cases were observed (Table 2).

Symptoms of abnormal behaviors including uncontrollable crying, shouting, panic, disorganized speech or aphasia, dazing, laughing, headbanging, body rocking, etc. In patients with abnormal behaviors, three crying, two shouting, six disorganized speech or aphasia, two dazing and two laughing cases were observed (Table 2).

With the exception of unresponsiveness, patients usually showed multiple symptoms (same category or different categories). Therefore, the number of abnormal events observed is slightly greater than the number of patients.

### Duration of Symptoms

**Figure 2:**
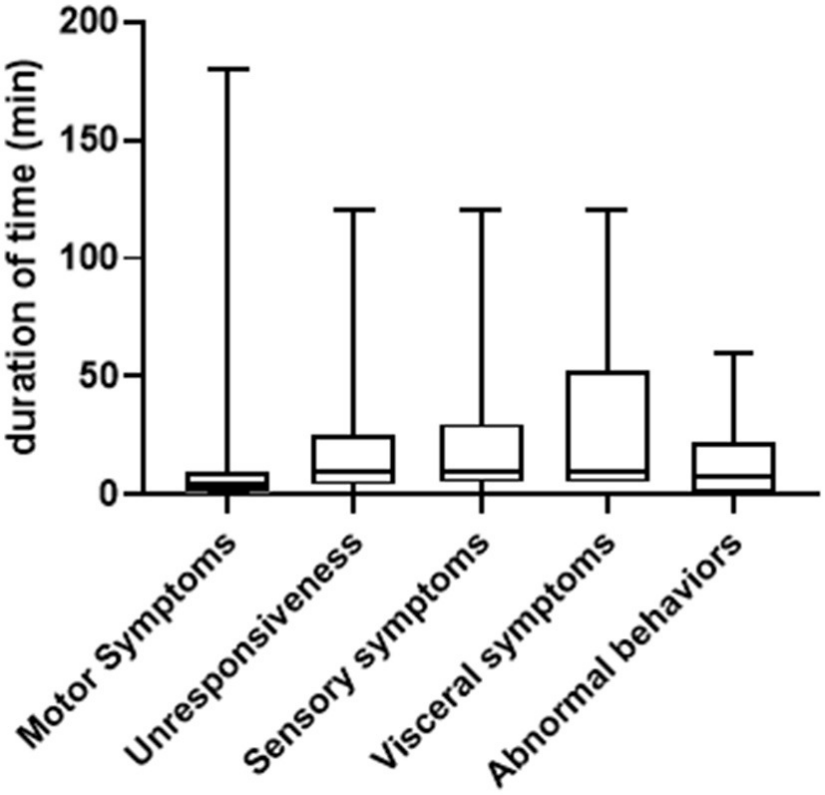
Comparison of shortest duration of time among difference type of symptoms (minute).

**Figure 3:**
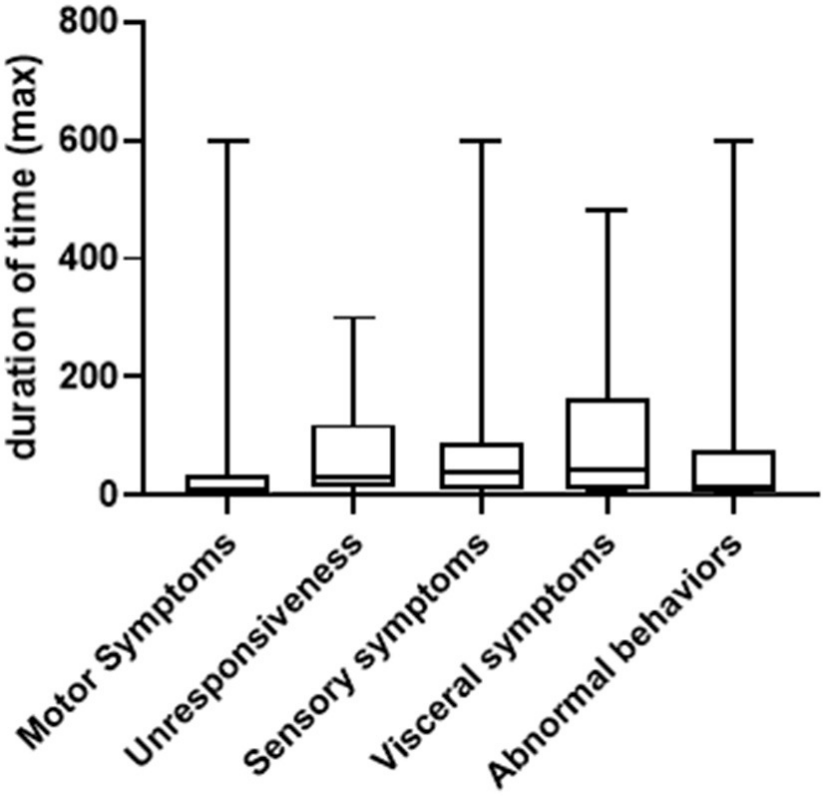
Comparison of longest duration of time among difference type of symptoms (minute).

In general, the duration of symptoms in PNES patients varied greatly, ranging from seconds to several hours. We summarized both the shortest and longest duration of time for each type of symptom. As shown in Figure 2, for the shortest duration of time, the range is between 10 s to 180 min (median = 4, 25% Percentile = 1, 75% Percentile = 10) for motor symptoms (patient to patient, same below); 30 s to 120 min (median = 10, 25% Percentile=4, 75% Percentile = 25) for unresponsiveness; 5 s to 120 min (median = 10, 25% Percentile = 5, 75% Percentile = 30) for sensory symptoms; 5–120 min (median = 10, 25% Percentile = 5, 75% Percentile = 52.5) for visceral symptoms and 10 s to 60 min (median = 7.5, 25% Percentile = 0.8, 75% Percentile = 22.5) for abnormal behaviors. For the longest duration of time, the range is between one to 300 min (median = 30, 25% Percentile = 12.5, 75% Percentile = 120) for motor symptoms; 5 s to 300 min (median = 10, 25% Percentile = 2, 75% Percentile = 35) for unresponsiveness, 0.5–600 min (median = 40, 25% Percentile = 10, 75% Percentile = 90) for sensory symptoms, 5 to 480 min (median = 45, 25% Percentile = 11.25, 75% Percentile = 165) for visceral symptoms and three to 600 min (median = 15, 25% Percentile = 5, 75% Percentile = 75) for abnormal behaviors (Figure 3).

### Frequency of Symptoms

To analyze the frequency of symptoms of each semiology category, patients were classified into three levels: mild, moderate, and severe, and were summarized in Table 3. Specifically, mild indicates an averagely long timespan between two symptom onsets (the time interval is equal or longer than 1 month), moderate indicates that the time interval is between 1 day and 1 month, severe indicates that the time interval is shorter than 24 h. As shown, most symptoms were in Severe grade (80 events, 60%), followed by Moderate (33 events, 23%) and Mild (21 events, 15%).

**Table 3:** Distribution of symptom frequency.

	**Mild**	**Moderate**	**Severe**	**Unknown**	**Total**
Motor symptoms	9	13	33	0	55
Unresponsiveness	3	8	14	0	25
Sensory symptoms	5	8	26	0	39
Visceral symptoms	2	1	5	1	9
Abnormal behaviors	2	3	9	1	15
All categories (events)	21	33	87	2	143
All categories (%)	15	23	60	1	100

*PNES symptoms of each semiology category was further classified into 4 groups basing on their frequency. Mild: time interval no less than 1 month; Moderate: time interval between 1 day and 1 month; Severe: time interval less than 24 h and Unknown. Events were calculated as indicated*.

### Risk Factors

Risk factors for PNES were analyzed based on medical records and were classified into two categories: persistence factors and predisposing factors (15). Persistence factors include environmental influences from family and/or school, to which children have been exposed for a long time. Predisposing factors, on the other side, referred to the direct trigger to the onset of current symptoms and/or hospitalization.

According to medical records, eight of eighty-eight patients had unclear triggers. For the remaining eighty patients, fifty-seven had only persistence factors, of which seven patients had two factors and only one factor was identified for the rest fifty patients. Specifically, poor parent-child relationship, foster care (raised by grandparents or other relatives than parents) were the most frequent persistence factors (Table 4).

**Table 4:** Risk factor analysis.

**Type of factors**	**Number of patients**
Only persistence factors	57
Poor patient-child relationship	29
Foster care	23
Dissatisfaction with daily life	12
Only predisposing factors	17
Mood swings	10
Being criticized	8
Fighting with others	6
Death of family members	3
Parents' leave	3
Injury	2
Arguments with others,	2
Illness	1
Both predisposing and persistence factors	6
Poor patient-child relationship	6
Foster care	5
Mood swings	3
Death of family members	1
Unknown	8
Total	88

*Risk factors of PNES analyzed basing on medical records and patient interviews*.

For the remaining twenty-three patients, seventeen were identified to have only predisposing factors. Mood swings, being criticized, and fighting with others were the top three most frequent predisposing factors. Death of family members, parents' leave, injury, arguments with others, and illness were other predisposing factors identified. Six patients had both persistent and predisposing factors (Table 4).

### Time Between Onset of Symptoms and Diagnosis

According to medical records, the median time between the onset of symptoms and diagnosis was 90 days, with the interquartile range of 212 days (7 months), the shortest diagnostic delay is 1 day and the longest diagnostic delay is 2,190 days (6 years).

### Comorbidity and Medical History

Twenty-three of eighty-eight patients had comorbidities (26%), of which epilepsy (five cases) and dysarthria (two cases) were the most common symptoms. Three patients showed subclinical epileptiform discharge and were previously misdiagnosed as epilepsy in other hospitals.

Forty-eight patients had taken or were taking AEDs by the time of visiting (55%), of which valproic acid was the most common drug and was followed by levetiracetam.

## Discussion

As a common type of paroxysmal disorder caused by psychological factors, PNES was reported prevalently affect the pediatric population. However, PNES was often misdiagnosed and mistreated as epilepsy as those two syndromes have many similar clinical features (16, 17). In this study, we reviewed the medical and VEEG records of 88 PNES cases from 4,268 pediatric patients with epilepsy like symptoms encountered in the past 6 years, semiological classification was done based on a comprehensive analysis of large sample data, the duration and frequency of PNES symptom onset as well as risk factors were also examined. These results would provide valuable insights into the diagnosis of PNES in children.

To distinguish PNES from epilepsy, clinical symptoms and VEEG are both necessary. Epilepsy is caused by abnormal brain electrical activity and patients' symptoms are usually correlated with neurophysiological events (like electric discharge), while this is absent in PNES (18–20). Therefore, it is applicable to diagnose without VEEG when patients with only simple symptoms. For complex symptoms, on the other hand, VEEG is required to exclude the involvement of abnormal electrical activity in the brain.

Of 4,268 patients with epilepsy like symptoms, 88 were diagnosed as PNES, the percentage is around 2%, which is substantially lower than previous reports (8–10, 21), it is possibly due to the fact that only inpatient cases were included in our study, therefore it does not reflect the true morbidity in the general population. In all PNES cases, 61% were boys, which is also inconsistent with previous studies in adults (22). Generally, women were more likely to develop PNES than men, perhaps due to extra social influences on women (23). However, in studies focusing on younger patients, an equivalent percentage between both genders was reported (24), or the percentage even higher in boys than girls, which is consistent with our results (25). For the ages of first symptoms onset, what we found is consistent with previous reports (9, 10, 26). Meanwhile, with the lowest age of 5 years old of PNES patients in our study, it should be particularly careful of patients with first symptom onset at an early age. The percentage of patients from rural area is higher than the one from urban area, which was not reported before. It is possibly due to the location of our hospital that patients from rural area are more like to visit, while with the lack of information regarding general population living in/out of the urban area, this need to be further addressed in future studies. The percentage of patients who had siblings (or from multiple children families) were higher than the one who didn't (or from only child families), while the underlying reason is still unclear. Overall, more studies are required to identify risk factors affecting PNES.

Although a series of semiological classifications have been established to analyzing the clinical features of pediatric PNES in the previous studies, according to our clinical practice in China, they were either incomplete or not easy to follow (8, 10, 13). For our semiological analysis, PNES related symptoms were classified into five categories: motor symptoms, unresponsiveness, sensory symptoms, visceral symptoms, and abnormal behaviors. PNES patients with motor symptoms were often misdiagnosed as epilepsy, while others found that the initiation and progression of motor symptoms in PNES were different from that in epileptic seizures (27). Besides, VEEG was also impacted by “shaking artifacts” in PNES patients (28). In the previous semiological classifications, rolling, shaking, pelvic movement, and arched back were classified into “movements,” which were classified and termed as motor symptoms in our setting. Another common symptom of PNES was unresponsiveness, patients with this symptom would suddenly fall or transient loss of consciousness (pseudosyncope). Once occurred, the involvement of hypoglycemia, hypotension, hyperammonemia, cardiogenic disease, coma, or other factors was examined and excluded. VEEG showed typical occipital alpha waves in PNES patients with unresponsiveness (mostly in elder patients), indicated they were in an awake state, which was consistent with previous reports (29–31). Sensory symptom is another group of symptoms in our study. Although similar symptoms were reported in PNES patients previously, the semiology of sensory symptom has not been fully characterized yet (8, 10, 13). Typical symptoms in this category including headache, dizziness, numbness of limbs, paroxysmal unclear vision, etc., therefore, the involvement/effect of migraine, vascular syncope, transient ischemic attack, hypertension, visual papilledema, or other possible triggers should be examined and excluded. Visceral symptoms including abdominal pain, abdominal discomfort, nausea, vomiting, and sometimes chest pain. For this type of symptoms, pathological factors should be first examined and excluded. Another clinical feature of PNES patients with visceral pain is that those patients usually had two or more different symptoms, so it is easier to distinguish from visceral symptoms caused by physiological factors. Abnormal behaviors including crying, shouting, laughing as well as other uncontrollable behaviors observed in our study. In reported semiological classifications for PNES, such kind of symptoms were considered as emotional or psychological disorders (32, 33), meanwhile, they were re-defined in our study with respect to behaviors rather than triggers. Importantly, as abnormal behaviors were also seen in immune encephalitis patients (34), the involvement of immune encephalitis should be examined for better diagnosis. Cerebrospinal fluid (CSF) examination is a commonly used strategy for patients who were suspected to have immune encephalitis (35). If CSF results indicate that immune encephalitis was not involved and VEEG shows normal background waves rather than slow waves featured in encephalitis (36), PNES would be the most likely cause. Overall, our study provides an alternative framework for semiological classification based on clinical features from Chinese pediatric patients.

Regarding the duration of symptoms, most PNES patients in our study showed long durations, which is different from the duration of epileptic seizures in other studies (typically did not exceed 5 min) (37, 38). For the frequency, most patients experienced multiple times of symptom onsets within days. In this case, duration and frequency could be considered as additional indicators of PNES diagnosis in children.

To identify potential psychological factors affecting PNES, medical records and self-reports were analyzed and factors were classified into two groups: persistence factors and predisposing factors. Our study shows that among persistence factors, influences from families were more prevalent than the ones from school, which is distinct from previous studies of pediatric patients in the United States and Italy (12, 39). Our results suggest that geographical and/or cultural differences may be critical in determining the importance of certain risk factors.

It was reported that the average time interval between symptom onsets and diagnosis was 7–10 years (40). Another report also showed that the length of delayed diagnosis in pediatric patients is from several weeks to 3.5 years (41). Misdiagnosis as epilepsy was responsible for most delayed cases, lacking VEEG and the unwillingness of patients' parents to visit were also accountable (42, 43). Our study showed that the median time for delayed diagnosis was 3 months, with the longest time of delayed diagnosis of 6 years, which were shorter than what was reported previously.

Previous studies have shown that a large proportion of children with PNES also had epilepsy (4). Besides, it was also proposed that people with epilepsy were more likely to develop PNES, mainly due to physiological and psychological influences including patients' past experiences (44). Our study supports the idea that epilepsy is the most frequent comorbidity in PNES and is possibly misdiagnosed with PNES. Critically, all symptoms in our study were observed when patients were awake, which was consistent with previous studies (29–31); besides, it was also reported that the “sleeping” state in which symptoms occurred in PNES patients were actually pseudo-sleep before onset. On the contrary, most epileptic seizure occurred when patients truly fall asleep (45). Therefore, the sleep/awake state could be considered as another indicator in diagnosis.

## Conclusion

In our study, medical and VEEG records from 88 pediatric PNES patients in China were retrospectively analyzed. The semiological analysis revealed that motor symptoms were predominant in all clinical symptoms. The duration of PNES symptoms was substantially longer than epilepsy, and most patients experienced multiple symptom onsets within a day. The influence of families is the most important risk factor in PNES development. Overall, our study provides insightful information regarding the clinical features and risk factors of pediatric PNES patients in one tertiary center in China, which would be valuable for future research and clinical practice. However, our study has several limitations: this is a study conducted in one center instead of a multicentric study, therefore, some of our findings cannot be generalized. Besides, a psychiatric evaluation was not performed, therefore, it is not possible to determine the presence and frequency of coexisting psychiatric disorders.

## Data Availability Statement

The raw data supporting the conclusions of this article will be made available by the authors, without undue reservation.

## Ethics Statement

The studies involving human participants were reviewed and approved by Xuanwu Hospital, Capital Medical University. Written informed consent to participate in this study was provided by the participants' legal guardian/next of kin.

## Author Contributions

L-PZ and Y-PW were the major contributors in writing the manuscript. YJ contributed to the design of the study. HH and D-WL contributed to the data analysis of all patients. L-PZ and Y-PW contributed to checking the manuscript. All authors read and approved the final manuscript.

## Conflict of Interest

The authors declare that the research was conducted in the absence of any commercial or financial relationships that could be construed as a potential conflict of interest.
